# Supplementary material for: The interactome of intact mitochondria by cross-linking mass spectrometry provides evidence for coexisting respiratory supercomplexes
Source: Mol Cell Proteomics. 2017 Dec 8;17(2):216–32. doi: 10.1074/mcp.RA117.000470 (PMC5795388; doi:10.1074/mcp.RA117.000470)
Supplement: Supplemental Data [file supp_RA117.000470_133922_0_supp_23978_fzffwf.zip › spectra_annotation/mito_DR_spectra_annotation/136-1-7-1-94-1.pdf]

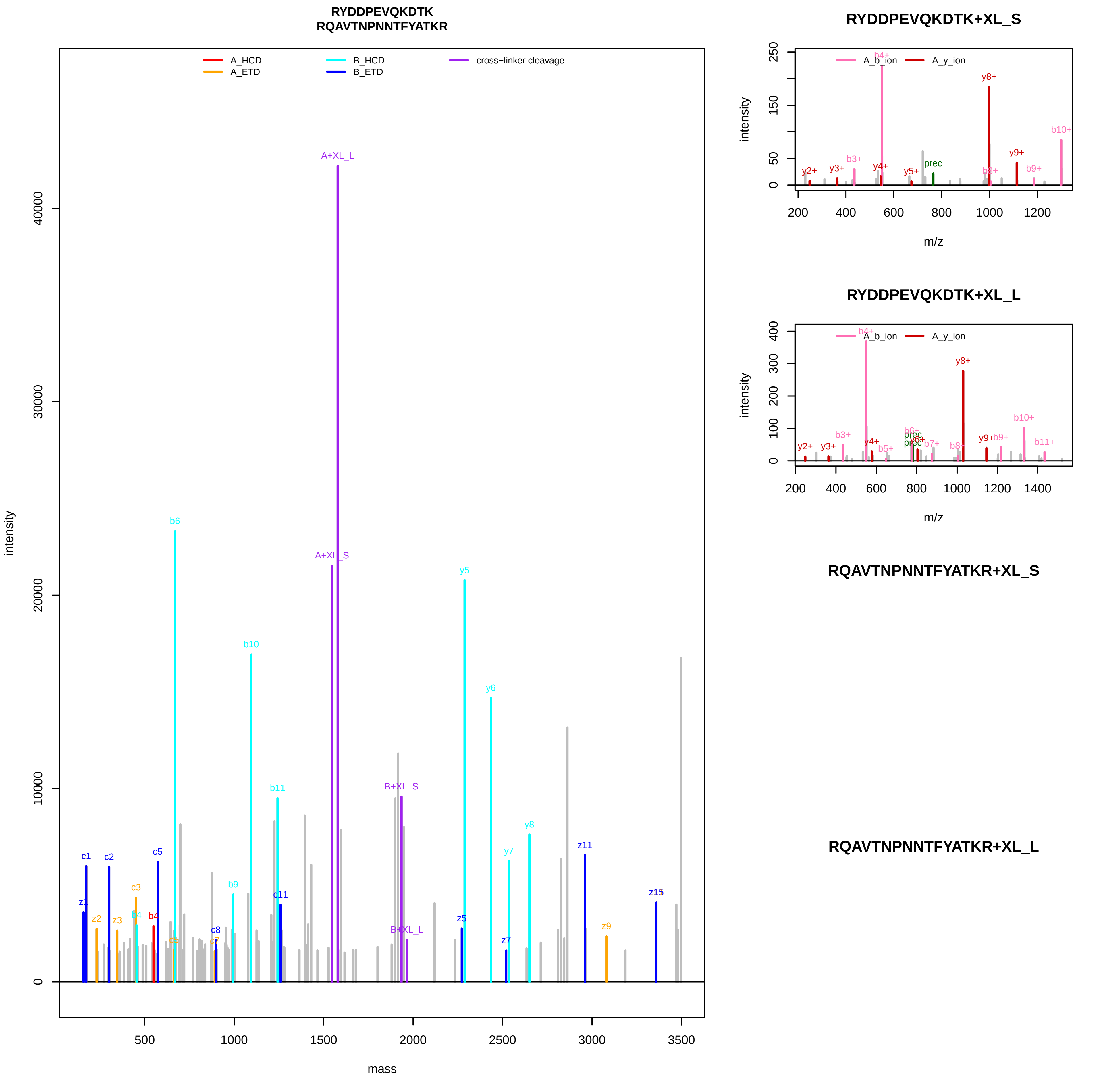
RYDDPEVQKDTK
RQAVTNPNNTFYATKR
A_HCD
A_ETD
B_HCD
B_ETD
cross−linker cleavage
A+XL_L
A+XL_S
B+XL_S
B+XL_L
b6
b10
b11
b9
b4
y5
y6
y7
y8
c1
c2
c3
c5
c7
c8
c11
z1
z2
z3
z5
z7
z9
z11
z15
intensity
mass
0
10000
20000
30000
40000
500
1000
1500
2000
2500
3000
3500
RYDDPEVQKDTK+XL_S
A_b_ion
A_y_ion
b3+
b4+
b8+
b9+
b10+
y2+
y3+
y4+
y5+
y8+
y9+
prec
intensity
m/z
0
50
150
250
200
400
600
800
1000
1200
RYDDPEVQKDTK+XL_L
A_b_ion
A_y_ion
b3+
b4+
b5+
b6+
b7+
b8+
b9+
b10+
b11+
y2+
y3+
y4+
y6+
y8+
y9+
prec
intensity
m/z
0
100
200
300
400
200
400
600
800
1000
1200
1400
RQAVTNPNNTFYATKR+XL_S
RQAVTNPNNTFYATKR+XL_L
